# Synthesis and Evaluation of a Series of 2-Substituted-5-Thiopropylpiperazine (Piperidine)-1,3,4-Oxadiazoles Derivatives as Atypical Antipsychotics

**DOI:** 10.1371/journal.pone.0035186

**Published:** 2012-04-30

**Authors:** Yin Chen, Xiangqing Xu, Xin Liu, Minquan Yu, Bi-Feng Liu, Guisen Zhang

**Affiliations:** 1 Department of Systems Biology, Huazhong University of Science and Technology, Wuhan, China; 2 Jiangsu Nhwa Pharmaceutical Co., Ltd., Xuzhou, Jiangsu, China; Baylor College of Medicine, United States of America

## Abstract

**Background:**

It is important to develop novel antipsychotics that can effectively treat schizophrenia with minor side-effects. The aim of our work is to develop novel antipsychotics that act on dopamine D_2_ and D_3_, serotonin 5-HT_1A_ and 5-HT_2A_ receptors with low affinity for the serotonin 5-HT_2C_ and H_1_ receptors, which can effectively cure positive symptoms, negative symptoms and cognitive impairment without the weight gain side-effect.

**Methodology/Principal Findings:**

A series of 2-substituted-5-thiopropylpiperazine (piperidine) -1,3,4-oxadiazoles derivatives have been synthesized and the target compounds were evaluated for binding affinities to D_2_, 5-HT_1A_ and 5-HT_2A_ receptors. Preliminary results indicated that compounds 14, 16 and 22 exhibited high affinities to D_2_, 5-HT_1A_ and 5-HT_2A_ receptors among these compounds. Further binding tests showed that compound 22 had high affinity for D_3_ receptor, and low affinity for serotonin 5-HT_2C_ and H_1_ receptors. In addition, compound 22 inhibited apomorphine-induced climbing behavior and MK-801-induced hyperactivity with no extrapyramidal symptoms liability in mice. Moreover, compound 22 exhibited acceptable pharmacokinetic properties.

**Conclusions/Significance:**

Compound 22 showed an atypical antipsychotic activity without liability for extrapyramidal symptoms. We anticipate compound 22 to be useful for developing a novel class of drug for the treatment of schizophrenia.

## Introduction

**Figure 1:**
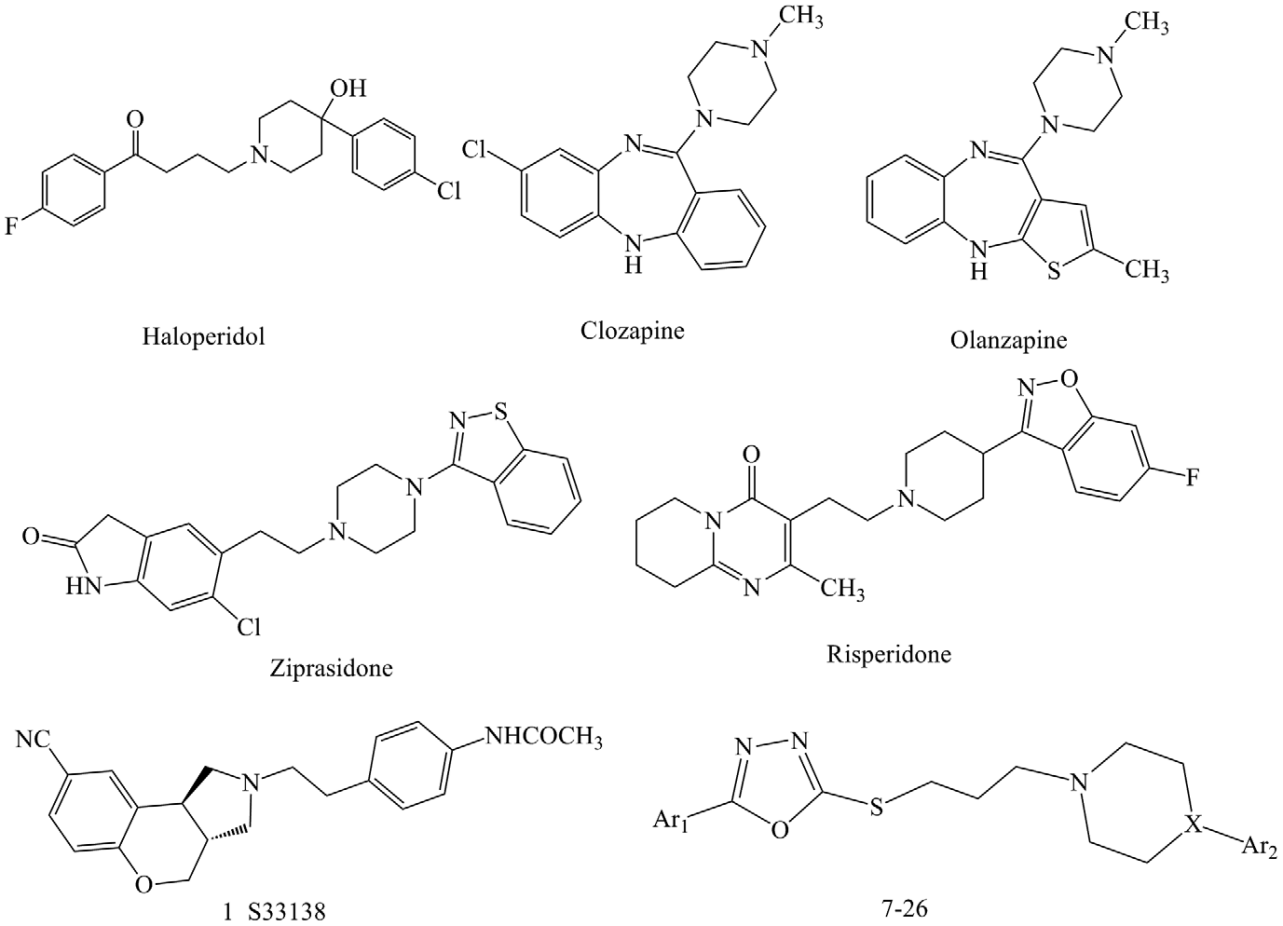
Title and reference compounds.

Schizophrenia is a serious mental disorder that significantly compromises the quality of life of those suffering from it. The early agents for the treatment of psychosis, the “typical” antipsychotics (haloperidol, Figure 1), were therapies for the positive symptoms of schizophrenia, but they failed to manage its negative symptoms and cognitive impairment [1]. Nevertheless, typical antipsychotics carry heavy side effects such as extrapyramidal symptoms (EPS) and hyperprolactinemia [2] [3] [4].

A breakthrough in the pharmacotherapy of schizophrenia was achieved by the introduction of the “atypical” antipsychotics (e.g., clozapine, ziprasidone, risperidone, quetiapine and olanzapine) which combines a potent antagonism for serotonin 5-HT_2A_ with a dopamine D_2_ receptors blockade [5]. A major advantage of atypical antipsychotics is their effectiveness in suppressing negative and cognitive symptoms [6] [7] [8] [9] [10]. However, it has been proved that atypical antipsychotics cause numerous side effects, such as substantial weight gain and QT interval prolongation [11] [12] [13]. Therefore, the discovery of novel antipsychotic agents that are effective and free of side effects with different chemical structures remains a challenging.

In the past decade, experimental evidence suggested that a complex binding profile is linked to the clinical efficacy of antipsychotic drugs. Indeed, the importance of designing multi-target G-protein-coupled receptors to deal with schizophrenia has been pointed out by many authors [14] [15] [16]. The 5-HT_1A_ receptor plays crucial roles in regulating psychoemotional, cognitive and motor functions in the central nervous system [17] [18]. Many relevant preclinical data suggested that 5-HT_1A_ receptor activation may contribute to the improved activity of certain atypical antipsychotic drugs, such as treatment cognitive and negative symptoms, and decrease the development of EPS in schizophrenia [19]. Blockade of D_2_ receptor was the key mechanism for controlling positive symptoms of schizophrenia [20]. The localization of D_3_ receptor in the limbic regions of brain suggests that this receptor subtype may be a target for developing antipsychotics, and thus, some works suggested that D_3_ antagonism may improve cognition [21] and reduce the risk of causing extrapyramidal side effect [22]. Compound S33138 (1) was shown to be a potent and selective dopamine D_3_ receptor antagonist,which has been in Phase IIb clinical trials for schizophrenia [23]. Furthermore, two or more receptors may be involved in the weight gain associated with the treatment of schizophrenia *via* atypical antipsychotic drugs. Blockade of H_1_ receptor by antipsychotics is more likely to be the primary cause of these adverse reactions [24] [25]. Although 5-HT_2C_ receptor blockade has been reported to counteract dopamine D_2_-mediated extrapyramidal side-effects (EPS) [26] and may also confer anxiolytic/antidepressant properties [27], 5-HT_2C_ receptor may be involved in the risk of obesity under chronic treatment [10] [28] [29]. Thus,the aim of our work is to develop a novel antipsychotic that acts on dopamine D_2_ and D_3_, serotonin 5-HT_1A_ and 5-HT_2A_ receptors with low affinity for the serotonin 5-HT_2C_ and H_1_ receptors, so that it could effectively cure positive symptoms, negative symptoms and cognitive impairment without the weight gain side-effect.

In fact, some of the latest efforts in the development of novel antipsychotic drugs are aimed at obtaining compounds with binding affinities for a certain number of receptors [10] [30] [31] [32]. To validate this multireceptor affinity profile approach to antipsychotics and to achieve an optimum interaction with dopamine and serotonin receptors, in this work, we report the synthesis and pharmacological evaluation of a new class of antipsychotic agents with a 1,3,4-oxadiazole system linked to the arylpiperazine (piperidine) group, which is one of the important kind of drugs for CNS-activity [33] [34] [35]. This strategy led to the synthesis of compounds 7–26 (Figure. 1) that allowed us to understand the SAR (structure-activity relationship) and to evaluate the pharmacological efficacy. The target compounds were subjected to preliminary pharmacological evaluation to determine their affinities for D_2_, D_3_, 5-HT_1A_, 5-HT_2A_, 5-HT_2C_ and H_1_ receptors. Among the derivatives prepared, compound 22 exhibited high affinity to D_2_, D_3,_ 5-HT_1A_ and 5-HT_2A_ receptors, with low affinity for 5-HT_2C_ and H_1_ receptors. In addition, Compound 22 inhibited apomorphine-induced climbing behavior and MK-801-induced hyperactivity without causing catalepsy in mice. In particular, compound 22 was more potent than clozapine.

## Results and Discussion

### Synthesis of Compounds 7–26

**Figure 2:**
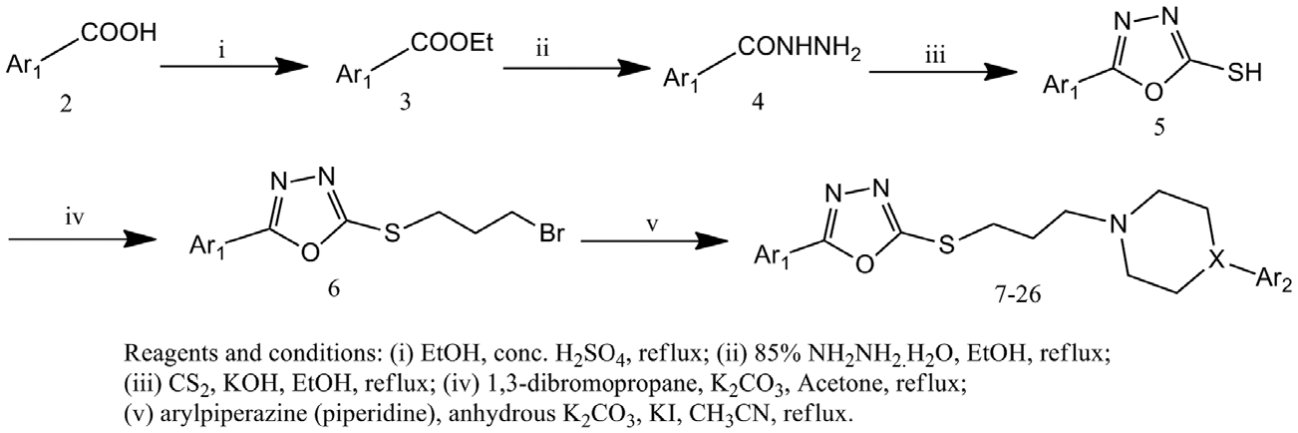
Synthesis of compounds 7–26.

The general strategy for the synthesis of compounds 7–26 was summarized in Figure 2. Aromatic acids 2 were esterified with absolute ethanol using conc. sulfuric acid as catalyst and the resulting esters 3 were refluxed with hydrazine hydrate in ethanol to give aroyl hydrazines 4. The acid hydrazides were then subjected to cyclisation with carbon disulphide in the presence of potassium hydroxide in absolute alcohol to afford the corresponding 5-aryl-1,3,4-oxadiazol-2-thiones (5). Compounds 5 reacted with 1,3-dibromopropane, in acetone to give 6. Compounds 6 reacted with an arylpiperazine (piperidine) in acetonitrile, in the presence of K_2_CO_3_ and a catalytic amount of potassium iodide, to give compounds 7–26 (Table 1) with good yields.

**Table 1:** Structure of compounds 7–26.

Compound	Ar_1_	X	Ar_2_	Mol.formula
7	Ph	N	2,3-di-CH_3_-Ph	C_23_H_28_N_4_OS
8	Ph	N	2-OCH_3_-Ph	C_22_H_26_N_4_O_2_S
9	Ph	N	2,3-di-Cl-Ph	C_21_H_22_Cl_2_N_4_OS
10	Ph	N	3-CF_3_-Ph	C_22_H_23_F_3_N_4_OS
11	Ph	N	benzo[d]isothiazole	C_22_H_23_N_5_OS_2_
12	Ph	CH	6-fluoro-benzo[d]isoxazole	C_22_H_23_FN_4_O_2_S
13	4-OCH_3_-Ph	N	benzo[d]isothiazole	C_23_H_25_N_5_O_2_S_2_
14	4-OCH_3_-Ph	CH	6-fluoro-benzo[d]isoxazole	C_24_H_25_FN_4_O_3_S
15	4-Cl-Ph	N	benzo[d]isothiazole	C_22_H_22_ClN_5_OS_2_
16	4-Cl-Ph	CH	6-fluoro-benzo[d]isoxazole	C_23_H_22_ClFN_4_O_2_S
17	4-CF_3_-Ph	N	benzo[d]isothiazole	C_23_H_22_F_3_N_5_OS_2_
18	4-CF_3_-Ph	CH	6-fluoro-benzo[d]isoxazole	C_24_H_22_F_4_N_4_O_2_S
19	4-F-Ph	N	benzo[d]isothiazole	C_22_H_22_FN_5_OS_2_
20	4-F-Ph	CH	6-fluoro-benzo[d]isoxazole	C_23_H_22_F_2_N_4_O_2_S
21	1-naphthalene	N	benzo[d]isothiazole	C_26_H_25_N_5_OS_2_
22	1-naphthalene	CH	6-fluoro-benzo[d]isoxazole	C_27_H_25_FN_4_O_2_S
23	2-thiophene	N	benzo[d]isothiazole	C_20_H_21_N_5_OS_3_
24	2-thiophene	CH	6-fluoro-benzo[d]isoxazole	C_21_H_21_FN_4_O_2_S_2_
25	4-pyridine	N	benzo[d]isothiazole	C_21_H_22_N_6_OS_2_
26	4-pyridine	CH	6-fluoro-benzo[d]isoxazole	C_22_H_22_FN_5_O_2_S

### *In vitro* studies of New Compounds

Initially, we investigated the effect of different amine moieties (Table 2, compounds 7–12) present in several established CNS agents: arylpiperazines such as N-(2,3-dichlorophenyl)-piperazine present in aripiprazole, and a substituted piperidine (N-(6-fluoro-benzisoxazol-3-yl)piperidine present in risperidone. According to Table 2, compounds 7–10 (phenylpiperazines) showed weak affinities for D_2_, 5-HT_1A_ and 5-HT_2A_. It should be noted that compounds 11 [(benzo[d]isothiazol-3-yl)piperazine] and 12 [(6-fluorobenzo[d]isoxazol-3-yl)piperidine] displayed high affinities for 5-HT_1A_ and 5-HT_2A_ receptors, and increased D_2_ receptor affinity in comparison to compounds 7–10.

**Table 2:** Binding affinities for D_2_, D_3_, 5-HT_1A_, 5-HT_2A_, 5-HT_2C_ and H_1_ receptors of compounds 7–26 and reference antipsychotics.^a^

Compound	Binding data of compounds, Ki±SEM (nM)					
	D_2_	5HT_1A_	5HT_2A_	D_3_	5HT_2C_	H_1_
7	>10000^b^	>10000^b^	373.9±33.0	–	–	–
8	>10000^b^	>10000^b^	>10000^b^	–	–	–
9	>10000^b^	>10000^b^	>10000^b^	–	–	–
10	>10000^b^	>10000^b^	>10000^b^	–	–	–
11	2568.5±321.3	19.0±2.3	61.4±5.8	–	–	–
12	2968.5±381.1	18.9±1.6	11.3±1.2	–	–	–
13	96.7±9.5	>10000	17.2±1.6	–	–	–
14	14.8±1.8	6.8±0.7	0.22±0.02	218.6±35.2	19.4±98.6	13.2±3.4
15	125.8±13.5	14.2±1.3	27.2±2.8	–	–	–
16	18.3±1.6	10.5±1.2	6.6±0.8	15.5±1.9	501.5±56.2	18.9±2.1
17	487.2±46.3	>10000	50.8±5.3	–	–	–
18	94.5±8.9	51.0±4.9	24.7±2.8	–	–	–
19	>10000	>10000	>10000	–	–	–
20	125.3±12.4	14.8±1.5	15.8±1.3	–	–	–
21	137.5±13.6	345.8±35.9	113.9±12.1	–	–	–
22	23.0±2.6	4.6±0.8	1.1±0.1	7.7±0.6	860.7±86.2	>10000^b^
23	>10000	449.6±49.8	54.1±6.9	–	–	–
24	91.0±11.2	96.6±10.3	50.3±6.5	–	–	–
25	>10000	>10000	389.8±36.5	–	–	–
26	260.3±32.1	60.6±8.6	59.2±7.6	–	–	–
risperidone	3.7±0.3	180±15	0.18±0.02	9.7±0.9	14.5±2.2	21.7±2.7
clozapine	128.7±1.9	141.6±1.6	11.6±1.3	239.8±29.6	16.2±2.7	3.8±0.5

a Ki values are taken from three experiments, expressed as means ±SEM.

b The Ki values were not calculated because the inhibition percentages at 10 µM were too low.

In order to improve D_2_ receptor affinity, further studies were conducted by introducing flouro, chloro, trifluoromethyl and methoxy substituents on the phenyl of the Ar_1_ (Table 2, compounds 13–20). When amine moiety Ar_2_ was (benzo[d]isothiazol-3-yl)piperazie (compounds 13, 15, 17 and 19), the order of affinities for the D_2_ and 5-HT_2A_ receptors was OCH_3_>Cl>CF_3_>F, and the high affinity for the 5-HT_1A_ substituents with Cl, but the affinities for 5-HT_1A_ receptor were obviously decreased when substituents with OCH_3_, CF_3_ and F. When amine moiety Ar_2_ was (6-fluorobenzo[d]isoxazol-3-yl) piperidine (compounds 14, 16, 18 and 20), the affinities order for the D_2_ receptor was OCH_3_>Cl>CF_3_>F, and the affinities order for the 5-HT_1A_ and 5-HT_2A_ receptors was OCH_3_>Cl>F>CF_3_. Compounds 14 and 16 exhibited high affinities for D_2_ (compound 14, Ki = 14.8 nM; compound 16, Ki = 18.3 nM), 5-HT_1A_ (compound 14, Ki = 6.8 nM; compound 16, Ki = 10.5 nM) and 5-HT_2A_ (compound 14, Ki = 0.22 nM; compound 16, Ki = 6.6 nM). Moreover, compounds 14 and 16 had higher affinities for all the three receptors than clozapine (D_2_, Ki = 128.7 nM; 5-HT_1A_, Ki = 141.6 nM; 5-HT_2A_, Ki = 11.6 nM). In particular, compounds 14 and 16 displayed higher affinities to 5-HT_1A_ receptor than risperidone (Ki = 180 nM). 5-HT_1A_ receptor implicated in the therapeutic efficacy of atypical antipsychotic drugs in treating the negative symptoms of schizophrenia and decreased the development of EPS in schizophrenia [18]. These results also indicated that compounds bearing a 6-fluorobenzo[d]isoxazol-3-yl)piperidine moiety (14, 16, 18 and 20) showed higher affinities to all three receptors than those with a (benzo[d]isothiazol-3-yl)piperazine fragment (13, 15, 17 and 19).

Furthermore, we investigated the effect of replacement of the Ar_1_ phenyl ring with naphthalene and heterocyclic (Table 2, compounds 21–26). These results indicated that compounds 21 and 22 with naphthalene showed good affinities for D_2_, 5-HT_1A_ and 5-HT_2A_ receptors. For example, compound 22 (D_2_, Ki = 23.0 nM; 5-HT_1A_, Ki = 4.6 nM; 5-HT_2A_, Ki = 1.1 nM) showed higher affinities than clozapine (D_2_, Ki = 128.7 nM; 5-HT_1A_, Ki = 141.6 nM; 5-HT_2A_, Ki = 11.6 nM). Moreover, compound 22 displayed higher affinity to 5-HT_1A_ receptor than risperidone (Ki = 180 nM). However, the introduction of an aromatic heterocycle at Ar_1_ (compounds 23–26) resulted in dramatic decrease of affinities for all the three receptors. These results pointed out the importance of the phenyl ring (Ar_1_) for the affinities at the D_2_, 5-HT_1A_ and 5-HT_2A_ receptors.

In line with the multiple receptor-targeting approaches for the development of new antipsychotic agents, compounds 14, 16, and 22 were selected for further binding tests to D_3_, 5-HT_2C_ and H_1_ receptors because they had high affinities for D_2_, 5-HT_1A_ and 5-H̀_A_ receptors. Previously, the D_3_ receptor was proposed for atypical antipsychotic drugs, and various pharmacological studies suggested that D_3_ antagonism might improve cognitive symptoms [21] and reduce catalepsy [22]. Results showed that compounds 14, 16 and 22 displayed higher affinities to D_3_ receptor than clozapine (Ki = 239.8 nM). In particular, compound 22 (Ki = 7.7 nM) displayed higher affinity than risperidone (Ki = 9.7 nM). Thus, these results suggested that compounds 14, 16 and 22 could reduce catalepsy in schizophrenia.

Treatment of schizophrenia with atypical antipsychotic drugs has been associated with weight gain. Two receptors, histamine H_1_ and 5-HT_2C_, have been suggested to be involved in this adverse event [24] [25] [10] [28] [29]. Several literatures have demonstrated that there is significant correlation between affinity for H_1_ receptor and weight gain [24] [25]. As shown in Table 2, compound 22 had much lower affinity (Ki>10000 nM) for H_1_ receptor than risperidone (Ki = 21.7 nM) and clozapine (Ki = 3.8 nM). Moreover, compound 22 had lower affinity to the 5-HT_2C_ receptor (Ki>500 nM) in comparison to risperidone (Ki = 14.5 nM) and clozapine (Ki = 16.2 nM). These results suggested that compound 22 exhibited a low potential to elicit treatment-caused weight gain.

### Acute Toxicity

The above results led to the conclusion that compound 22 exhibited high affinity for dopamine D_2_ and D_3_, serotonin 5-HT_1A_ and 5-HT_2A_ receptors, with low affinity for the serotonin 5-HT_2C_ and H_1_ receptors. We then assayed the acute toxicity of the new compound by determining their LD_50_ value. Compound 22 showed good safety profiles even at the highest dose tested (LD_50_>2000 mg/kg).

**Table 3:** In *vivo* pharmacological profile of compound 22. Inhibition of different behavioral responses after oral administration of the test and reference Compounds.

Compound	Apomorphine- induced climbing (ED_50_, mg/kg, po, A)	MK-801-induced hyperactivity (ED_50_, mg/kg, po, B)	CAT (catalepsy) (ED_50_ mg/kg,po, C)	C/A	C/B
22	3.68 (2.79–4.86)^a^	3.58 (2.59–8.28)	>300	81.52	83.80
risperidone	0.02 (0.014–0.024)	0.01 (0.004–1.51)	0.3	15	30
clozapine	7.99 (7.34–8.69)	5.06 (3.41–9.69)	92.73	11.61	18.33
haloperidol	0.09 (0.054–0.177)	0.19 (0.15–0.26)	0.22	2.44	1.16

a 95% Confidence limits given in parentheses.

### *In vivo* Studies

An initial behavioral screening was performed on compound 22 based on their multiple receptors affinity profile. The atypical antipsychotics have been used for relieving positive symptoms at doses without EPS [10]. In this study, the side-effect liability was evaluated by the horizontal bar test, which is very sensitive for catalepsy induced by dopamine D_2_ receptor blockade [10]. Antipsychotic potential of these compounds were assessed by apomorphine-induced climbing and dizocilpine (MK-801) induced hyperactivity. Apomorphine-induced climbing was potently reduced by D_2_ receptor antagonists [36], while selective antagonism of the effect of the noncompetitive N-methyl-D-aspartate (NMDA) antagonist MK-801 had been proposed as a robust animal model for the negative and cognitive symptoms of schizophrenia [37].

The apomorphine-induced climbing model is based on the induction of a hyperdopaminergic state by apomorphine. This model has been classically linked to motor agitation and one of the schizophrenia positive symptoms [36]. In the apomorphine-induced climbing model, compound 22 produced the significant reversal of apomorphine-induced climbing, with ED_50_ value of 3.68 mg/kg (Table 3). In comparison, risperidone, clozapine and haloperidol produced reversal of apomorphine-induced climbing with ED_50_ values of 0.02, 7.99 and 0.09 mg/kg, respectively. These results suggested that compound 22 was slightly more potent at blocking the D_2_ receptors in vivo than clozapine. This was also consistent with their estimated Ki values at the D_2_ receptor.

The MK-801-induced hyperactivity model has been used to indirectly evaluate the ability of compounds to oppose cortical dopaminergic hypofunction induced by NMDA receptor blockade [37]. In this test, compound 22 significantly inhibited MK-801-induced hyperactivity with ED_50_ value of 3.58 mg/kg (Table 3). In comparison, risperidone, clozapine and haloperidol yielded ED_50_ values of 0.01, 5.06 and 0.19 mg/kg, respectively. These results indicated that compound 22 was more potent than clozapine.

Catalepsy is often used as the method for predicting the incidence of extrapyramidal motor disorders. In this model (Table 3), it was clear that haloperidol had the highest propensity to induce catalepsy (ED_50_ 0.22 mg/kg), in agreement with the high capacity of this drug to block D_2_ receptor [38]. In contrast, compound 22 exhibited a low potential to induce catalepsy with ED_50_ value>300 mg/kg (Table 3), similar to those of risperidone and clozapine (ED_50_ risperidone 0.3 mg/kg, clozapine 92.73 mg/kg). Moreover, these results suggested that the therapeutic indices of compound 22 calculated between their efficacy (apomorphine or MK-801 models) and side effects (catalepsy) were in the range 81–83, while the therapeutic indices of risperidone and clozapine were roughly 11–30. Thus, in contrast to risperidone and clozapine, compound 22 had a high threshold for inducing catalepsy which might, by analogy, translate into lower clinical EPS liability.

Overall, compound 22 significantly inhibited apomorphine-induced climbing behavior and MK-801-induced hyperactivity without causing catalepsy. These results suggested a preferential ability of compound 22 to modulate mesolimbic instead of nigrostriatal dopaminergic neurotransmission, highlighting their atypicality and low propensity to induce unwanted extrapyramidal motor disturbances at therapeutically useful doses.

### Pharmacokinetic Properties of Compound 22

Compound 22 was selected based on its in *vitro* profile for in *vivo* characterization. Table 4 highlights the pharmacokinetic parameters of compound 22 in the rat using both intravenous and oral administration. Intravenous administration of compound 22 to rats (5 mg/kg, n = 6) resulted in detectable plasma levels (half-life (t_1/2_) = 9.3 h), and oral administration of compound 22 to rats (20 mg/kg, n = 6) resulted in a t_1/2_ of 8.6 h. The area under the curve (AUC) value of compound 22 was 6239.0 ng×h/mL after intravenous administration versus 13602.7 ng×h/mL after oral administration. The C_max_ value after oral dosing was 723.6 ng/mL, and the T_max_ value was 5.0 h. The bioavailability of compound 22 was 54.5%.

**Table 4:** Plasma pharmacokinetic data following administration of compound 22 (i.v. dose of 5 mg/kg and p.o. dose of 20 mg/kg) in rats (n = 6/group).

Route	C_max_ (ng/mL)	T_max_ (h)	t_1/2_ (h)	AUC_0-inf_ (ng×h/mL)	F (%)
oral	723.6	5	8.6	13602.7	54.5
iv	–	–	9.3	6239.0	

In summary, we described the synthesis and pharmacological evaluation of a series of 2-substituted-5-thiopropylpiperazine (piperidine)-1,3,4-oxadiazoles derivatives as potential multi-target antipsychotics. Among the derivatives synthesized, compound 22 showed high affinity for dopamine D_2_ and D_3_, serotonin 5-HT_1A_ and 5-HT_2A_ receptors, with low affinity for the serotonin 5-HT_2C_ and H_1_ receptors. In *vivo* animal models showed that compound 22 had high potential for treating symptoms of schizophrenia without causing catalepsy. Moreover, compound 22 exhibited acceptable pharmacokinetic properties.

## Materials and Methods

### Synthesis of Compounds 7–26

Melting points were determined in open capillary tubes and are uncorrected. ^1^H NMR spectra were recorded at 400 MHz on a Varian Inova Unity 200 spectrometer in CDCl_3_ solution. Chemical shifts were given in δ values (ppm), using tetramethylsilane (TMS) as the internal standard; coupling constants (J) were given in Hz. Signal multiplicities were characterized as s (singlet), d (doublet), t (triplet), q (quartet), m (multiplet), br (broad signal). Reagents were all of analytical grade or of chemical purity. Analytical TLC was performed on silica gel GF254. Column chromatographic purification was carried out using silica gel.

#### General procedure for the synthesis of aroyl hydrazines 4 (a–h) [39]

A mixture of aromatic acids 2 (10 mmol), ethanol (20 mL) and a catalytic amount of conc. H_2_SO_4_ were refluxed for 3 h. The reaction mixture was cooled and the formed solids were filtered to give ester 3, which was refluxed with 85% hydrazine hydrate (10 mL) in ethanol (20 mL) for 2 h. After completion of the reaction by TLC, the reaction mixture was cooled and the formed solids were filtered and washed with chilled ethanol (1 mL) to give the corresponding aroyl hydrazines 4 (a–h).

benzohydrazide (4a). Yield: 88%; mp: 109–111°C (lit [40], mp: 111–113°C).

4-methoxybenzohydrazide (4b). Yield: 85%; mp: 133–135°C (lit [41], mp: 135–137°C).

4-chlorobenzohydrazide (4c). Yield: 82%; mp: 115–117°C (lit [41], mp: 117–118°C).

4-(trifluoromethyl)benzohydrazide (4d). Yield: 87%; mp:114–116°C (lit [40], mp:115–116°C).

4-fluorobenzohydrazide (4e). Yield: 80%; mp: 159–162°C (lit [41], mp: 160–163°C).

1-naphthohydrazide (4f). Yield: 80%; mp: 161–163°C (lit [41], mp: 160–163°C).

thiophene-2-carbohydrazide (4g). Yield: 84%; mp: 136–138°C (lit [42], mp: 135–137°C).

isonicotinohydrazide (4h). Yield: 84%; mp: 170–171°C (lit [43], mp: 172°C).

#### General procedure for the preparation of 5-aryl-1,3,4-oxadiazol-2-thiones 5 (a–h) [44]

A mixture of 10 mmol of potassium hydroxide, 10 mmol of compounds 4 (a–h), and 15 mmol of carbon disulfide in 50 mL of absolute ethanol was refluxed for 8 h. After the solvent was evaporated in vacuum, the residue was dissolved in ice-cold water and acidified with dilute hydrochloric acid. The precipitate was filtered off, washed with water, and recrystallized from absolute ethanol to give compounds 5 (a–h).

5-phenyl-1,3,4-oxadiazole-2-thione (5a). Yield: 87%; mp: 215–217°C (lit [45], mp: 218°C); MS (ESI) m/z 178 (M^+^).

5-(4-methoxyphenyl)-1,3,4-oxadiazole-2-thione (5b). Yield: 71%; mp: 201–203°C (lit [45], mp: 204°C); MS (ESI) m/z 208 (M^+^).

5-(4-chlorophenyl)-1,3,4-oxadiazole-2-thione (5c). Yield: 79%; mp: 175–177°C (lit [45], mp: 175°C); MS (ESI) m/z 212 (M^+^).

5-(4-(trifluoromethyl)phenyl)-1,3,4-oxadiazole-2-thione (5d). Yield: 72%; mp: 168–170°C; MS (ESI) m/z 246 (M^+^).

5-(4-fluorophenyl)-1,3,4-oxadiazole-2-thione (5e). Yield: 85%; mp: 205–207°C (lit [46]. mp: 208–209°C); MS (ESI) m/z 196 (M^+^).

5-(naphthalen-1-yl)-1,3,4-oxadiazole-2-thione (5f). Yield:71%; mp: 198–200°C; MS (ESI) m/z 228 (M^+^).

5-(thiophen-2-yl)-1,3,4-oxadiazole-2-thione (5g). Yield: 65%; mp: 199–201°C (lit [47], mp: 201–203°C); MS (ESI) m/z 184 (M^+^).

5-(pyridin-4-yl)-1,3,4-oxadiazole-2-thione (5h). Yield: 55%; mp: 270–271°C (lit [48], mp: 272–272.5°C; MS (ESI) m/z 179 (M^+^).

#### General procedure for the preparation of 5-aryl-2-((3-bromopropyl)thio)-1,3,4-oxadiazole 6 (a–h)

1,3-dibromopropane (3 mmol) was added to a solution of compounds 5 (a–h) (1 mmol) and potassium carbonate in acetone (50 mL), and the mixture was refluxed for 3 h. The progress of the reaction was monitored by TLC. After cooling to room temperature, the mixture was filtered, the solvent was evaporated and the residue was recrystallized from hexane/EtOH to yield compounds 6 (a-h).

2-((3-bromopropyl)thio)-5-phenyl-1,3,4-oxadiazole (6a) : Yield: 79.1%; mp: 55–57°C. ^1^H-NMR (CDCl_3_) δ 2.33–2.39 (m, 2H), 3.46 (t, 2H, J = 13.6 Hz), 3.73 (t, 2H, J = 12.4 Hz), 7.47–7.53 (m, 3H), 7.99–8.02 (m, 2H).

2-((3-bromopropyl)thio)-5-(4-methoxyphenyl)-1,3,4-oxadiazole (6b) : Yield: 67.2%; mp: 65–67°C. ^1^H-NMR (CDCl_3_) δ 2.32–2.38 (m, 2H), 3.44 (t, 2H, J = 14 Hz), 3.72 (t, 2H, J = 12.4 Hz), 3.87 (s, 3H), 6.98–7.01 (m, 2H), 7.93–7.96 (m, 2H).

2-((3-bromopropyl)thio)-5-(4-chlorophenyl)-1,3,4-oxadiazole (6c) : Yield: 71.1%; mp: 98–100°C. ^1^H-NMR (CDCl_3_) δ 2.34–2.37 (m, 2H), 3.46 (t, 2H, J = 13.6 Hz), 3.72 (t, 2H, J = 12.4 Hz), 7.48 (d, 2H, J = 8.4 Hz), 7.94 (d, 2H, J = 8.4 Hz).

2-((3-bromopropyl)thio)-5-(4-fluorophenyl)-1,3,4-oxadiazole (6d) : Yield: 74.7%; mp: 88–90°C. ^1^H-NMR (CDCl_3_) δ 2.41–2.46 (m, 2H), 3.46 (t, 2H, J = 13.6 Hz), 3.58 (t, 2H, J = 12.4 Hz), 7.21–7.22 (m, 2H), 8.00–8.03 (m, 2H).

2-((3-bromopropyl)thio)-5-(4-(trifluoromethyl)phenyl)-1,3,4-oxadiazole (6e) : Yield: 70.1%; mp: 83–85°C.^ 1^H-NMR (CDCl_3_) δ 2.42–2.48 (m, 2H), 3.49 (t, 2H, J = 13.6 Hz), 3.58 (t, 2H, J = 12.4 Hz), 7.77 (d, 2H, J = 8.4 Hz), 8.13 (d, 2H, J = 8.4 Hz).

2-((3-bromopropyl)thio)-5-(naphthalen-1-yl)-1,3,4-oxadiazole (6f) : Yield: 72.8%; mp: 85–87°C. ^1^H-NMR (CDCl_3_) δ 2.47–2.51 (m, 2H), 3.51 (t, 2H, J = 14 Hz), 3.61 (t, 2H, J = 12.4 Hz), 7.55–7.68 (m, 3H), 7.93 (d, 1H, J = 8 Hz), 8.03 (d, 1H, J = 8 Hz), 8.13 (d, 1H, J = 7.2 Hz), 9.20 (d, 1H, J = 8.4 Hz).

2-((3-bromopropyl)thio)-5-(pyridin-4-yl)-1,3,4-oxadiazole (6g) : Yield: 68.7%; mp: 75–77°C. ^1^H-NMR (CDCl_3_) δ 2.42–2.48 (m, 2H), 3.50 (t, 2H, J = 13.6 Hz), 3.58 (t, 2H, J = 12.4 Hz), 7.86–7.88 (m, 2H), 8.80–8.82 (m, 2H).

2-((3-bromopropyl)thio)-5-(thiophen-2-yl)-1,3,4-oxadiazole (6h) : Yield: 72.5%; mp: 95–97°C. ^1^H-NMR (CDCl_3_) δ 2.40–2.45 (m, 2H), 3.44 (t, 2H, J = 13.6 Hz), 3.57 (t, 2H, J = 12.4 Hz), 7.15–7.17 (m, 1H), 7.54–7.55 (m, 1H), 7.70–7.72 (m, 1H).

#### General procedure for the preparation of compounds 7–26

To a suspension of compounds 6 (0.32 mmol) and K_2_CO_3_ (1.22 mmol) in acetonitrile (5.0 mL), arylpiperazine (piperidine) (0.32 mmol) and a catalytic amount of KI were added and the resulting mixture was refluxed for 12 h. After filtering, the resulting filtrate was evaporated to dryness under reduced pressure. The residue was suspended in water (10.0 ml) and extracted with dichloromethane (3×25 mL). The combined organic layers were evaporated under reduced pressure, and the crude product was purified by means of chromatography (5% MeOH/CHCl_3_) to yield compounds 7–26.

2-((3-(4-(2,3-dimethylphenyl)piperazin-1-yl)propyl)thio)-5-phenyl-1,3,4-oxadiazole (7): Yield: 69.3%; mp: 86–88°C. ^1^H-NMR (CDCl_3_) δ 2.08 (m, 2H), 2.21 (s, 3H), 2.26 (s, 3H), 2.58 (m, 6H), 2.90 (m, 4H), 3.39 (m, 2H), 6.88–6.90 (m, 2H), 7.06 (m, 1H), 7.50 (m, 3H), 7.99–8.01 (m, 2H). MS (ESI) m/z 409.2 ([M+H]^+^).

2-((3-(4-(2-methoxyphenyl)piperazin-1-yl)propyl)thio)-5-phenyl-1,3,4-oxadiazole (8).

Yield: 78.6%; oil. ^1^H-NMR (CDCl_3_) δ 2.08–2.15 (m, 2H), 2.60–2.70 (m, 6H), 3.12 (br, 4H), 3.42 (t, 2H, J = 14 Hz), 3.88 (s, 3H), 6.88–7.04 (m, 4H), 7.50–7.55 (m, 3H), 8.03–8.05 (m, 2H). MS (ESI) m/z 411.2 ([M+H]^+^).

2-((3-(4-(2,3-dichlorophenyl)piperazin-1-yl)propyl)thio)-5-phenyl-1,3,4-oxadiazole (9): Yield: 75.3%; mp: 82–84°C. ^1^H-NMR (CDCl_3_) δ 2.06–2.13 (m, 2H), 2.58–2.66 (m, 6H), 3.07 (br, 4H), 3.39 (t, 2H, J = 14.4 Hz), 6.93–6.95 (m, 1H), 7.11–7.16 (m, 2H), 7.47–7.53 (m, 3H), 7.99–8.02 (m, 2H). MS (ESI) m/z 449.1 ([M+H]^+^).

2-((3-(4-(3-(trifluoromethyl)phenyl)piperazin-1-yl)propyl)thio)- 5-phenyl-1,3,4-oxadiazole.

(10): Yield: 67.2%; mp: 68–69°C. ^1^H-NMR (CDCl_3_) δ 2.06–2.13 (m, 2H), 2.56–2.64 (m, 6H), 3.24 (t, 4H, J = 10 Hz), 3.39 (t, 2H, J = 14.4 Hz), 7.04–7.10 (m, 3H), 7.34 (t, 1H, J = 16 Hz), 7.47–7.53 (m, 3H), 7.99–8.02 (m, 2H). MS (ESI) m/z 449.2 ([M+H]^+^).

2-((3-(4-(benzo[d]isothiazol-3-yl)piperazin-1-yl)propyl)thio)-5-phenyl-1,3,4-oxadiazole (11): Yield: 69.1%; mp: 69–71°C. ^1^H-NMR (CDCl_3_) δ 2.11–2.14 (m, 2H), 2.64 (t, 2H, J = 13.6 Hz), 2.72–2.74 (m, 4H), 3.40 (t, 2H, J = 14 Hz), 3.58–3.60 (m, 4H), 7.33–7.37 (m, 1H), 7.44–7.52 (m, 4H), 7.80 (d, 1H, J = 8 Hz), 7.89 (d, 1H, J = 8.4 Hz), 7.99 (d, 2H, J = 2 Hz). MS (ESI) m/z 438.2 ([M+H]^+^).

2-(3-(4-(6-fluorobenzo[d]isoxazol-3-yl)piperidin-1-yl)propylthio)-5-phenyl-1,3,4-oxadiazole (12): Yield: 81.6%; mp: 106–107°C. ^1^H-NMR (CDCl_3_) δ 2.05–2.18 (m, 8H), 2.57 (t, 2H, J = 13.6 Hz), 3.05–3.09 (m, 3H), 3.40 (t, 2H, J = 14.4Hz), 7.05 (m, 1H), 7.24 (dd, 1H, J_1_ = 2 Hz, J_2_ = 2 Hz), 7.49–7.51 (m, 3H), 7.68–7.71 (m, 1H), 7.99–8.02 (m, 2H). MS (ESI) m/z 439.2 ([M+H]^+^).

2-((3-(4-(benzo[d]isothiazol-3-yl)piperazin-1-yl)propyl)thio)-5-(4-methoxyphenyl)-1,3,4-.

oxadiazole (13): Yield: 70.6%; mp: 88–89°C. ^1^H-NMR (CDCl_3_) δ 2.08–2.11 (m, 2H), 2.61 (t, 2H, J = 13.6 Hz), 2.69 (t, 4H, J = 9.6 Hz), 3.38 (t, 2H, J = 14.4 Hz), 3.57 (t, 4H, J = 9.6 Hz), 3.87 (s, 3H), 6.99 (d, 2H, J = 8.8 Hz), 7.35 (t, 1H, J = 8.4 Hz), 7.46 (t, 1H, J = 8.8 Hz), 7.80 (d, 1H, J = 8.4 Hz), 7.89–7.95 (m, 3H). MS (ESI) m/z 468.2 ([M+H]^+^).

2-(3-(4-(6-fluorobenzo[d]isoxazol-3-yl)piperidin-1-yl)propylthio)-5-(4-methoxyphenyl)-1,3,4-oxadiazole (14): Yield: 78.3%; mp: 103–104°C. ^1^H-NMR (CDCl_3_) δ 2.04–2.17 (m, 8H), 2.57 (t, 2H, J = 13.6 Hz), 3.04–3.08 (m, 3H), 3.37 (t, 2H, J = 14 Hz), 3.86 (s, 3H), 6.99–7.07 (m, 3H), 7.21–7.30 (m, 1H), 7.68–7.71 (m, 1H), 7.91–7.95 (m, 2H). MS (ESI) m/z 469.2 ([M+H]^+^).

2-((3-(4-(benzo[d]isothiazol-3-yl)piperazin-1-yl)propyl)thio)-5-(4-chlorophenyl)-1,3,4-.

oxadiazole (15): Yield: 79.8%; mp: 99–101°C. ^1^H-NMR (CDCl_3_) δ 2.08–2.12 (m, 2H), 2.59–2.71 (m, 6H), 3.40 (t, 2H, J = 14.4 Hz), 3.55–3.58 (m, 4H), 7.33–7.36 (m, 1H), 7.44–7.47 (m, 3H), 7.79–7.89 (m, 1H), 7.90–7.94 (m, 3H). MS (ESI) m/z 472.2 ([M+H]^+^).

2-(3-(4-(6-fluorobenzo[d]isoxazol-3-yl)piperidin-1-yl)propylthio)-5-(4-chlorophenyl)-1,3,4-.

oxadiazole (16): Yield: 80.3%; mp: 115–117°C. ^1^H-NMR (CDCl_3_) δ 2.08–2.20 (m, 8H), 2.56–2.59 (m, 2H), 3.06–3.09 (m, 3H), 3.42 (t, 2H, J = 14.4 Hz), 7.04–7.06 (m, 1H), 7.22–7.24 (m, 1H), 7.46–7.49 (m, 2H), 7.68–7.72 (m, 1H), 7.94–7.96 (m, 2H). MS (ESI) m/z 473.2 ([M+H]^+^).

2-((3-(4-(benzo[d]isothiazol-3-yl)piperazin-1-yl)propyl)thio)-5-(4-(trifluoromethyl)phenyl)-.

1,3,4-oxadiazole (17): Yield: 71.8%; mp: 106–107°C. ^1^H-NMR (CDCl_3_) δ 2.10–2.14 (m, 2H), 2.60–2.71 (m, 6H), 3.43 (t, 2H, J = 14 Hz), 3.58 (br, 4H), 7.33–7.37 (m, 1H), 7.44–7.48 (m, 1H), 7.75–7.82 (m, 3H), 7.90 (d, 1H, J = 8 Hz), 8.13 (d, 2H, J = 8 Hz). MS (ESI) m/z 506.2 ([M+H]^+^).

2-(3-(4-(6-fluorobenzo[d]isoxazol-3-yl)piperidin-1-yl)propylthio)-5-(4-(trifluoromethyl)phenyl)-1,3,4-oxadiazole (18): Yield: 68.4%; mp: 122–124°C. ^1^H-NMR (CDCl_3_) δ 2.08–2.19 (m, 8H), 2.59 (t, 2H, J = 13.6 Hz), 3.06–3.12 (m, 3H), 3.43 (t, 2H, J = 14 Hz), 7.06–7.08 (m, 1H), 7.23–7.27 (m, 2H), 7.68–7.72 (m, 1H), 7.77 (d, 2H, J = 8.4 Hz), 8.14 (d, 1H, J = 8 Hz). MS (ESI) m/z 507.2 ([M+H]^+^).

2-(3-(4-(benzo[d]isothiazol-3-yl)piperazin-1-yl)propylthio)-5-(4-fluorophenyl)-1,3,4-.

oxadiazole (19): Yield: 71.2%; mp: 89–91°C. ^1^H-NMR (CDCl_3_) δ 2.06–2.10 (m, 2H), 2.57–2.68 (m, 6H), 3.38 (t, 2H, J = 14 Hz), 3.53–3.56 (m, 4H), 7.13–7.17 (m, 2H), 7.32–7.43 (m, 2H), 7.77–7.99 (m, 4H). MS (ESI) m/z 456.2 ([M+H]^+^).

2-(3-(4-(6-fluorobenzo[d]isoxazol-3-yl)piperidin-1-yl)propylthio)-5-(4-fluorophenyl)-1,3,4-.

oxadiazole (20): Yield: 61.9%; mp: 118–120°C. ^1^H-NMR (CDCl_3_) δ 2.05–2.17 (m, 8H), 2.57 (t, 2H, J = 13.6 Hz), 3.05–3.07 (m, 3H), 3.40 (t, 2H, J = 14 Hz), 7.05–7.06 (m, 1H), 7.16–7.25 (m, 3H), 7.68–7.71 (m, 1H), 8.00–8.03 (m, 2H). MS (ESI) m/z 457.2 ([M+H]^+^).

2-(3-(4-(benzo[d]isothiazol-3-yl)piperazin-1-yl)propylthio)-5-(naphthalen-1-yl)-1,3,4-.

oxadiazole (21): Yield: 66.9%; oil. ^1^H-NMR (CDCl_3_) δ 2.11–2.18 (m, 2H), 2.62–2.72 (m, 6H), 3.44 (t, 2H, J = 14 Hz), 3.56–3.59 (m, 4H), 7.32–8.13 (m, 10H), 9.21 (d, 1H, J = 8.8 Hz). MS (ESI) m/z 488.3 ([M+H]^+^).

2-(3-(4-(6-fluorobenzo[d]isoxazol-3-yl)piperidin-1-yl)propylthio)-5-(naphthalen-1-yl)-1,3,4-.

oxadiazole (22): Yield: 76.8%; mp: 107–109°C. ^1^H-NMR (CDCl_3_) δ 2.06–2.16 (m, 8H), 2.60 (t, 2H, J = 6.8 Hz), 3.07–3.10 (m, 3H), 3.45 (t, 2H, J = 14 Hz), 7.04–7.06 (m, 1H), 7.23–7.26 (m, 1H), 7.56–7.59 (m, 2H), 7.67–7.69 (m, 2H), 7.92 (d, 1H, J = 8.4 Hz), 8.02 (d, 1H, J = 8 Hz), 8.13 (d, 1H, J = 7.2 Hz), 9.21 (d, 1H, J = 8.8 Hz). MS (ESI) m/z 489.3 ([M+H]^+^).

2-(3-(4-(benzo[d]isothiazol-3-yl)piperazin-1-yl)propylthio)-5-(thiophen-2-yl)-1,3,4-.

oxadiazole (23): Yield: 68.5%; mp: 65–67°C. ^1^H-NMR (CDCl_3_) δ 2.07–2.11 (m, 2H), 2.60 (t, 2H, J = 13.6 Hz), 2.68–2.70 (m, 4H), 3.38 (t, 2H, J = 14 Hz), 3.55–3.58 (m, 4H), 7.13–7.15 (m, 1H), 7.35 (m, 1H), 7.44–7.47 (m, 1H), 7.51–7.53 (m, 1H), 7.69–7.70 (m, 1H), 7.80 (d, 1H, J = 8 Hz), 7.90 (d, 1H, J = 8.4 Hz). MS (ESI) m/z 444.2 ([M+H]^+^).

2-(3-(4-(6-fluorobenzo[d]isoxazol-3-yl)piperidin-1-yl)propylthio)-5-(thiophen-2-yl)-1,3,4-.

oxadiazole (24): Yield: 60.3%; mp: 91–92°C. ^1^H-NMR (CDCl_3_) δ 2.04–2.17 (m, 8H), 2.56 (t, 2H, J = 13.6 Hz), 3.05–3.07 (m, 3H), 3.38 (t, 2H, J = 14.4 Hz), 7.05–7.08 (m, 1H), 7.14–7.16 (m, 1H), 7.22–7.25 (m, 1H), 7.52–7.54 (m, 1H), 7.68–7.71 (m, 2H). MS (ESI) m/z 445.2 ([M+H]^+^).

2-(3-(4-(benzo[d]isothiazol-3-yl)piperazin-1-yl)propylthio)-5-(pyridin-4-yl)-1,3,4-oxadiazole (25): Yield: 66.3%; mp: 93–94°C. ^1^H-NMR (CDCl_3_) δ 2.09–2.12 (m, 2H), 2.59–2.71 (m, 6H), 3.44 (t, 2H, J = 14 Hz), 3.56 (t, 4H, J = 9.6 Hz), 7.33–7.36 (m, 1H), 7.44–7.47 (m, 1H), 7.79–7.91 (m, 4H), 8.78–8.80 (m, 2H). MS (ESI) m/z 439.2 ([M+H]^+^).

2-(3-(4-(6-fluorobenzo[d]isoxazol-3-yl)piperidin-1-yl)propylthio)-5-(pyridin-4-yl)-1,3,4-.

oxadiazole (26): Yield: 71.2%; mp: 115–117 °C. ^1^H-NMR (CDCl_3_) δ 2.06–2.19 (m, 8H), 2.59 (t, 2H, J = 13.6 Hz), 3.06–3.10 (m, 3H), 3.44 (t, 2H, J = 14 Hz), 7.03–7.08 (m, 1H), 7.23–7.26 (m, 1H), 7.68–7.72 (m, 1H), 7.86–7.87 (m, 2H),8.79–8.81 (m, 2H). MS (ESI) m/z 440.2 ([M+H]^+^).

### Ethics Statement

Chinese Kun Ming (KM) Mice (20±2.0 g) and Sprague-Dawley (SD) rats (250±5.0 g) were used as experimental animals in this study. Animals were housed under standardized conditions for light and temperature and received standard rat chow and tap water and libitum. Animals were randomly assigned to different experimental groups and each group was kept in a separate cage. All the research involving animals in this study follows the guidelines of the byelaw of experiments on animals, and has been approved by the Ethics and Experimental Animal Committee of Jiangsu Nhwa Pharmaceutical Co., Ltd.

### *In Vitro* Binding Assays

#### General procedures

All the new compounds were dissolved in 5% DMSO. The following specific radioligands and tissue sources were used: (a) serotonin 5-HT_1A_ receptor, [^3^H]8-OH-DPAT, rat brain cortex; (b) serotonin 5-HT_2A_ receptor, [^3^H]ketanserin, rat brain cortex; (c) serotonin 5-HT_2C_ receptor, [^3^H]mesulergine, rat brain cortex; (d) dopamine D_2_ receptor, [^3^H]spiperone, rat striatum; (e) dopamine D_3_ receptor, [^3^H]spiperone, rat olfactory tubercle (f) histamine H_1_ receptor, [^3^H] pyrilamine, guinea pig cerebellum.

Total binding was determined in the absence of no-specific binding and compounds. Specific binding was determined in the presence of compounds. Non-specific binding was determined as the difference between total and specific binding.

Percentage of inhibition (%) = (total binding − specific binding)×100%/(total binding − nonspecific binding).

Blank experiments were carried out to determine the effect of 5% DMSO on the binding and no effects were observed. Compounds were tested at least three times over a 6 concentration range (10^−5^ M to 10^−10^ M), IC_50_ values were determined by nonlinear regression analysis using Hill equation curve fitting. Ki values were calculated based on the Cheng and Prussoff equation: Ki = IC_50_/(1+C/K_d_) where C represents the concentration of the hot ligand used and K_d_ its receptor dissociation constant were calculated for each labeled ligand. Mean Ki values and SEM were reported for at least three independent experiments.

#### 5-HT_1A_ binding assay [49]

Rat cerebral cortex was homogenized in 20 volumes of ice-cold Tris-HCl buffer (50 mM, pH 7.7) using an ULTRA TURAX homogeniser, and was then centrifuged at 32000 g for 10 min. The resulting pellet was then resuspended in the same buffer, incubated for 10 min at 37 °C, and centrifuged at 32000 g for 10 min. The final pellet was resuspended in Tris-HCl buffer containing 10 µM Pargyline, 4 mM CaCl_2_ and 0.1% ascorbic acid.

Total binding each assay tube was added 900 µL of the tissue suspension, 50 µL of 0.5 nM [^3^H]8-OH-DPAT (187.4 Ci/mmol, Perkin Elmer Life Sciences, Boston, MA, USA), 50 µL Tris-HCl buffer containing 10 µM Pargyline, 4 mM CaCl_2_ and 0.1% ascorbic acid.

Non-specific binding each assay tube was added 900 µL of the tissue suspension, 50 µL of 0.5 nM [^3^H]8-OH-DPAT, 50 µL of 10 µM serotonin.

Specific binding each assay tube was added 900 µL of the tissue suspension, 50 µL of 0.5 nM [^3^H]8-OH-DPAT, 50 µL of new compounds or reference drug.

The tubes were incubated at 37°C for 30 min. The incubation was followed by a rapid vacuum filtration through Whatman GF/B glass filters, and the filtrates were washed twice with 5mL cold buffer and transferred to scintillation vials. Scintillation fluid (3.0 mL) was added and the radioactivity bound was measured using a Beckman LS 6500 liquid scintillation counter.

#### 5-HT_2A_ binding assay [49]

Rat cerebral cortex was homogenized in 20 volumes of ice-cold Tris-HCl buffer (50 mM, pH 7.7) using an ULTRA TURAX homogeniser, and centrifuged at 32000 g for 20 min. The resulting pellet was resuspended in the same quantity of the buffer centrifuged for 20 min. The final pellet was resuspended in 50 volumes of the Tris-HCl buffer.

Total binding each assay tube was added 900 µL of the tissue suspension, 50 µL of 0.6 nM [^3^H]ketanserin (60.0 Ci/mmol, Perkin Elmer Life Sciences, Boston, MA, USA), 50 µL Tris-HCl buffer.

Non-specific binding each assay tube was added 900 µL of the tissue suspension, 50 µL of 0.6 nM [^3^H]ketanserin, 50 µL of 10 µM methisergide.

Specific binding each assay tube was added 900 µL of the tissue suspension, 50 µL of 0.6 nM [^3^H]ketanserin, 150 µL of new compounds or reference drug.

The tubes were incubated at 37°C for 15 min. The incubation was followed by a rapid vacuum filtration through Whatman GF/B glass filters, and the filtrates were washed twice with 5 mL cold buffer and transferred to scintillation vials. Scintillation fluid (3.0 mL) was added and the radioactivity bound was measured using a Beckman LS 6500 liquid scintillation counter.

#### 5-HT_2C_ binding assay [49]

Rat cerebral cortex was homogenized in 20 volumes of ice-cold Tris-HCl buffer (50 mM, pH7.7) using ULTRA TURAX homogeniser, and centrifuged at 32000 g for 20 min. The resulting pellet was resuspended in the same quantity of the buffer centrifuged for 20 min. The final pellet was resuspended in 50 volumes of the Tris-HCl buffer.

Total binding each assay tube was added 900 µL of the tissue suspension, 50 µL of 1 nM [^3^H]mesulergine (85.4 Ci/mmol; Perkin Elmer Life Sciences, Boston, MA, USA), 50 µL Tris-HCl buffer.

Non-specific binding each assay tube was added 900 µL of the tissue suspension, 50 µL of 1 nM [^3^H]mesulergine, 50 µL of 10 µM mianserin.

Specific binding each assay tube was added 900 µL of the tissue suspension, 50 µL of 1 nM [^3^H]mesulergine, 50 µL of new compounds or reference drug.

The tubes were incubated at 37°C for 15 min. The incubation was followed by a rapid vacuum filtration through Whatman GF/B glass filters, and the filtrates were washed twice with 5 mL cold buffer and transferred to scintillation vials. Scintillation fluid (3.0 mL) was added and the radioactivity bound was measured using a Beckman LS 6500 liquid scintillation counter.

#### D_2_ dopaminergic binding assay [49]

Rat striatum was homogenized in 20 volumes of ice-cold 50 mM Tris-HCl buffer (pH 7.7) using an ULTRA TURAX homogeniser, and centrifuged twice for 10 min at 48,000 g with resuspension of the pellet in fresh buffer. The final pellet was resuspended in 50 mM ice-cold Tris-HCl containing 120 mM NaCl, 5 mM KCl, 2 mM CaCl_2_, 1 mM MgCl_2_, 0.1% ascorbic acid and 5 µM pargyline.

Total binding each assay tube was added 900 µL of the tissue suspension, 50 µL of 0.5 nM [^3^H]spiperone (16.2 Ci/mmol; Perkin Elmer Life Sciences, Boston, MA, USA), 50 µL Tris-HCl buffer containing 120 mM NaCl, 5 mM KCl, 2 mM CaCl_2_, 1 mM MgCl_2_, 0.1% ascorbic acid and 5 µM pargyline.

Non-specific binding each assay tube was added 900 µL of the tissue suspension, 50 µL of 0.5 nM [^3^H]spiperone, 50 µL of 10 µM (+)-butaclamol.

Specific binding each assay tube was added 900 µL of the tissue suspension, 50 µL of 0.5 nM [^3^H]spiperone, 50 µL of new compounds or reference drug.

The tubes were incubated at 37°C for 15 min. The incubation was followed by a rapid vacuum filtration through Whatman GF/B glass filters, and the filtrates were washed twice with 5 mL cold buffer and transferred to scintillation vials. Scintillation fluid (3.0 mL) was added and the radioactivity bound was measured using a Beckman LS 6500 liquid scintillation counter.

### D_3_ Dopaminergic Binding Assay [37]

Rat olfactory tubercle was homogenized in 20 volumes of ice-cold 50 mM Hepes Na (pH 7.5) using an ULTRA TURAX homogeniser, and centrifuged twice for 10 min at 48,000 g with resuspension of the pellet in fresh buffer. The final pellet was resuspended in 50 mM Hepes Na, pH 7.5, containing 1 mM EDTA, 0.005% ascorbic acid, 0.1% albumin, 200 nM eliprodil.

Total binding each assay tube was added 900 µL of membranes, 50 µL of 0.6 nM [^3^H]spiperone (16.2 Ci/mmol; Perkin Elmer Life Sciences, Boston, MA, USA), 50 µL of 50 mM Hepes Na, pH 7.5, containing 1 mM EDTA, 0.005% ascorbic acid, 0.1% albumin, 200 nM eliprodil.

Non-specific binding each assay tube was added 900 µL of membranes, 50 µL of [^3^H]spiperone, 50 µL of 1 µM dopamine.

Specific binding each assay tube was added 900 µL of Membranes, 50 µL of [^3^H]spiperone, 50 µL of new compounds or reference drug.

The tubes were incubated at 25°C for 60 min. The incubation was followed by a rapid vacuum filtration through Whatman GF/B glass filters, and the filtrates were washed twice with 5 mL cold buffer and transferred to scintillation vials. Scintillation fluid (3.0 mL) was added and the radioactivity bound was measured using a Beckman LS 6500 liquid scintillation counter.

### Histamine H_1_ Binding Assay [50]

Guinea pig cerebellum was homogenized in 20 volumes of ice-cold 50 mM phosphate buffer (pH 7.4) using an ULTRA TURAX homogeniser, and centrifuged twice for 10 min at 50,000 g with resuspension of the pellet in fresh buffer. The final pellet was resuspended in phosphate buffer.

Total binding each assay tube was added 900 µL of membranes 50 µL of 1 nM [^3^H]pyrilamine (20.0 Ci/mmol; Perkin Elmer Life Sciences, Boston, MA, USA), 50 µL phosphate buffer.

Non-specific binding each assay tube was added 900 µL of membranes, 50 µL of [^3^H]pyrilamine, 50 µL of 1 µM promethazine.

Specific binding each assay tube was added 900 µL of Membranes, 50 µL of [^3^H]pyrilamine, 50 µL of new compounds or reference drug.

The tubes were incubated at 30°C for 60 min. The incubation was followed by a rapid vacuum filtration through Whatman GF/B glass filters, and the filtrates were washed twice with 5 mL cold buffer and transferred to scintillation vials. Scintillation fluid (3.0 mL) was added and the radioactivity bound was measured using a Beckman LS 6500 liquid scintillation counter.

#### Acute toxicity study

Mice (5 mice in each group) were orally dosed with increasing doses of the compound 22 (200, 500, 1000, 1500 and 2000 mg/kg). The number of surviving animals was recorded after 24 h of drug administration, and the percent mortality in each group was calculated. The LD_50_ value was calculated by using the program SPSS (Statistical Package for the Social Science).

#### MK-801-induced hyperactivity [51]

Mice (10 mice in each group) were orally dosed with vehicle or increasing doses of the haloperidol (0.06, 0.2, 0.6, 2.0 and 6 mg/kg), clozapine (1, 2.5, 7, 20 and 60 mg/kg), risperidone (0.01, 0.03, 0.1, 0.3 and 1.0 mg/kg) and compound 22 (3, 5, 10, 20 and 30 mg/kg). Animals were placed in Plexiglas cages for evaluating locomotor activity. After 30 min, the animals were challenged with 0.3 mg/kg (sc) of MK-801 and the locomotor activity of each animal was recorded for 90 min.

### Behavioral Tests

#### Apomorphine-induced climbing [36]

Mice (10 mice in each group) were orally dosed with vehicle or increasing doses of the haloperidol (0.1, 0.13, 0.17, 0.23 and 0.3 mg/kg), clozapine (4.0, 7.5, 9.5 and 12.5 mg/kg), risperidone (0.01, 0.03, 0.1 and 0.3 mg/kg), compound 22 (0.8, 2.5, 8, 25 and 80 mg/kg). Animals were then challenged at 30 minutes post-injection with 1.0 mg/kg of the apomorphine in 0.9% NaCl+0.1% ascorbic acid, placed in cylindrical wire cages (12 cm in diameter, 14 cm in height), and observed for climbing behavior at 10, 20 and 30 min post dose. The climbing behaviour was scored as follows: 3–4 paws on the cage floor = 0 score; 2 and 3 paws on the cage = 1 score; 4 paws on the cage = 2 score.

#### Catalepsy test [38]

Mice (10 mice in each group) were orally dosed with vehicle or increasing doses of the haloperidol (0.18, 0.35, 0.75, 1.5 and 3.0 mg/kg), clozapine (25, 50, 100, 150 and 200 mg/kg), risperidone (0.1, 0.6, 1.2, 2.5 and 5.0 mg/kg), compound 22 (50, 150 and 300 mg/kg). Catalepsy was evaluated on a metal bar 0.6 cm in diameter positioned 4.5 cm above the tabletop. The test consisted in positioning the animal with its forepaws on the bar and recording how long it remained hanging onto the bar; the end-point was 60 s and an all-or-none criterion was used.

#### Pharmacokinetics study in rat

The HPLC conditions were as follows: column, Shim-pack ODS 5.0 µm×150 mm×2.0 mm I.D (SHIMADZU, Japanese); mobile phase, 0.0167% HCOOH (TEDIA Company, USA)/acetonitrile (Merck Company, Germany)50/50; flow rate, 0.2 mL/min; column temperature, 40°C.

For routine compound 22 screening rats (n = 6/group) were dosed via the lateral tail vein at the indicated dose for intravenous administration (5 mg/kg, 100% saline) or via oral gavage (20 mg/kg, suspension in 0.5% methylcellulose). At 30 min, 1 h, 2 h, 3 h, 4 h, 5 h, 6 h, 7 h and 24 h after administration, serial blood samples were collected from the lateral tail vein into heparinized collection tubes (approximately 0.25 mL). The plasma was separated by centrifugation, and the sample was prepared for analysis HPLC/MS by protein precipitation with acetonitrile. The plasma samples were analyzed for drug and internal standard via HPLC-MS/MS protocol.

#### Statistics

To estimate the potency of test and reference compounds, the ED_50_ values and their 95% confidence limits were calculated by using the program SPSS (Statistical Package for the Social Science).

## References

[1] Lewine RRJ, Fogg L, Meltzer HY (1983). Assessment of Negative and Positive Symptoms in Schizophrenia.. Schizophr Bull.

[2] Marde SR, Wirshing WC, Van Putten T (1991). Drug treatment of schizophrenia. Overview of recent research.. Schizophr Res.

[3] Baldessarini RJ, Tarsy D (1980). Dopamine and the pathophysiology of dyskinesias induced by antipsychotic drugs.. Annu Rev Neurosci.

[4] Boyd AE, Reichlin S (1978). Neural control of prolactin secretion in man.. Psychoneuroendocrino.

[5] Campbell M, Young PI, Bateman DN (1999). The use of atypical antipsychotics in the management of schizophrenia.. Br J Clin Pharmacol.

[6] Jones CA, McCreary AC (2008). Serotonergic approaches in the development of novel antipsychotics.. Neuropharmacology.

[7] Shapiro D, Renock S, Arrington E, Chiodo L, Liu L (2003). Aripiprazole: a novel atypical antipsychotic drug with a unique and robust pharmacology.. Neuropsychopharmacol.

[8] Roth BL, Hanizavareh SM, Blum AE (2004). Serotonin receptors represent highly favorable molecular targets for cognitive enhancement in schizophrenia and other disorders.. Psychopharmacology.

[9] Meltzer HY, Li Z, Kaneda Y, Ichikawa J (2003). Serotonin receptors: their key role in drugs to treat schizophrenia.. Prog Neuropsychopharmacol Biol Psych.

[10] Butini S, Gemma S, Campiani G, Franceschini S, Trotta F (2009). Discovery of a new class of potential multifunctional atypical antipsychotic agents targeting dopamine D_3_ and serotonin 5-HT_1A_ and 5-HT_2A_ receptors: Design, synthesis, and effects on behavior.. J Med Chem.

[11] Tamminga CA (1997). The promise of new drugs for schizophrenia treatment.. Can J Psychiatry.

[12] Vohora D (2007). Atypical antipsychotic drugs: current issues of safety and efficacy in the management of schizophrenia.. Curr Opin Invest Drugs.

[13] Schultz SH, North SW, Shields CG (2007). Schizophrenia: a review.. Am Fam Physician.

[14] Meltzer HY (2004). What’s atypical about atypical antipsychotic drugs?. Curr Op Pharmacol.

[15] Morphy R, Rankovic Z (2009). Designing Multiple Ligands – Medicinal Chemistry Strategies and Challenges.. Curr Pharm Des.

[16] Wong EH, Tarazi FI, Shahid M (2010). The effectiveness of multi-target agents in schizophrenia and mood disorders: Relevance of receptor signature to clinical action.. Pharmacol Ther.

[17] Ohno Y (2010). New insight into the therapeutic role of 5-HT_1A_ receptors in central nervous system disorders.. Cent Nerv Syst Agents Med Chem.

[18] Politis M, Wu K, Loane C, Quinn NP, Brooks DJ (2010). Serotonergic neurons mediate dyskinesia side effects in Parkinson’s patients with neural transplants.. Sci Transl Med.

[19] Millan MJ (2000). Improving the Treatment of Schizophrenia: Focus on Serotonin (5-HT)1A Receptors.. J Pharmacol Exp Ther.

[20] Meltzer HY, Matsubara S, Lee MA (1989). Classification of typical and atypical antipsychotic drug on the basis of D_1_, D_2_ and serotonin 2 pK values.. J Pharmacol Exp Ther.

[21] Meltzer HY (2004). Cognitive factors in schizophrenia: causes, impact, and treatment.. CNS Spectrosc.

[22] Bézard E, Ferry S, Mach U, Stark H, Leriche L (2003). Attenuation of Levodopa-Induced Dyskinesia by Normalizing Dopamine D(3) Receptor Function.. Nat Med.

[23] Millan MJ, Loiseau F, Dekeyne A, Gobert A, Flik G (2008). S33138 (N-[4-[2-[(3aS,9bR)-8-cyano-1,3a,4,9b-tetrahydro[1] benzopyrano[3,4-c]pyrrol-2(3H)-yl)-ethyl]phenyl-acetamide), a Preferential Dopamine D_3_ versus D_2_ Receptor Antagonist and Potential Antipsychotic Agent: III. Actions in Models of Therapeutic Activity and Induction of Side Effects.. J Pharmacol Exper Ther.

[24] Kroeze WK, Hufeisen SJ, Popadak BA, Renock S, Steinberg S (2003). H_1_-histamine receptor affinity predicts short-term weight gain for typical and atypical antipsychotic drugs.. Neuropsychopharmacol.

[25] Kim SF, Huang AS, Snowman AD, Teuscher T, Snyder SH (2007). Antipsychotic Drug-Induced Weight Gain Mediated by Histamine H_1_ Receptor-Linked Activation of Hypothalamic AMP-Kinase.. Proc Natl Acad Sci USA.

[26] Reavill C, Kettle A, Holland V, Riley G, Blackburn TP (1999). : Attenuation of haloperidol-induced catalepsy by a 5-HT_2C_ receptor antagonist.. Br J Pharmacol.

[27] Wood MD, Reavill C, Trail B, Wilson A, Stean T (2001). SB-243213; a selective 5-HT_2C_ receptor inverse agonist with improved anxiolytic profile: Lack of tolerance and withdrawal anxiety.. Neuropharmacology.

[28] Buckland PR, Hoogendoorn B, Guy CA, Smith SK, Coleman SL (2005). Low gene expression conferred by association of an allele of the 5-HT_2C_ receptor gene with antipsychotic-induced weight gain. Am.. J. Psychiatry.

[29] Reynolds GP, Hill MJ, Kirk SL (2006). The 5-HT_2C_ receptor and antipsychoticinduced weight gain-mechanisms and genetics.. J Psychopharmacol.

[30] Garzya V, Forbes IT, Gribble AD, Hadley MS, Lightfoot AP (2007). Studies towards the identification of a new generation of atypical antipsychotic agents.. Bioorg Med Chem Lett.

[31] Ablordeppey SY, Altundas R, Bricker B, Zhu XY, Kumar EV (2008). Identification of a butyrophenone analog as a potential atypical antipsychotic agent: 4-[4-(4-Chlorophenyl)-1,4-diazepan-1-yl]-1-(4-fluorophenyl)butan-1-one.. Bioorg Med Chem.

[32] Neves G, Menegatti R, Antonio CB, Grazziottin LR, Vieira RO (2010). Searching for multi-target antipsychotics: Discovery of orally active heterocyclic N-phenylpiperazine ligands of D_2_-like and 5-HT_1A_ receptors.. Bioorg Med Chem.

[33] Glennon RA, Naiman RA, Lyon RA, Titeler M (1988). Arylpiperazine Derivatives as High-Affinity 5-HT_1A_ Serotonin Ligands.. J Med Chem.

[34] Lowe JA, Seeger TF, Nagel AA, Howard HR, Seymour PA (1991). 1-Naphthylpiperazine derivatives as potential atypical antipsychotic agents.. J Med Chem.

[35] Obniska J, Kolaczkowski M, Bojarski AJ, Duszyńska B (2006). Synthesis, anticonvulsant activity and 5-HT_1A_, 5-HT_2A_ receptor affinity of new N-[(4-arylpiperazin-1-yl)-alkyl] derivatives of 2-azaspiro[4.4]nonane and [4.5]decane-1,3-dione.. Eur J Med Chem.

[36] Costall B, Naylor RJ, Nohria V (1978). Climbing behaviour induced by apomorphine in mice: A potential model for the detection of neuroleptic activity.. Eur J Pharmacol.

[37] Campiani G, Butini S, Fattorusso C, Catalanotti B, Gemma S (2004). Pyrrolo[1,3]benzothiazepine-based serotonin and dopamine receptor antagonists. Molecular modeling, further structure-activity relationship studies, and identification of novel atypical antipsychotic agents.. J Med Chem.

[38] Xiberas X, Martinot JL, Mallet L, Artiges E, Loc’H C (2001). Extrastriatal and striatal D_2_ dopamine receptor blockade with haloperidol or new antipsychotic drugs in patients with schizophrenia.. Br J Psychiatry.

[39] Gaonkar SL, Rai KML, Prabhuswamy B (2006). Synthesis and antimicrobial studies of a new series of 2-{4-[2-(5-ethylpyridin-2-yl)ethoxy]phenyl}-5-substituted-1,3,4-oxadiazoles.. Eur J Med Chem.

[40] Doria JS, Fanny PB, Marina I, Mesquita PKF (2011). Novel benzofuroxan derivatives against multidrug-resistant Staphylococcus aureus strains: Design using Topliss decision tree, synthesis and biological.. Bioorg Med Chem.

[41] Li XR, Zhao ZG, Li GH, Shi PY (2010). Design and synthesis of novel molecular tweezer anion receptors based on diphenic acid carbonyl thiosemicarbazide.. J Chem Res (S).

[42] Abdel-Aziz M, Abuo-Rahma GD, Hassan AA (2009). Synthesis of novel pyrazole derivatives and evaluation of their antidepressant and anticonvulsant activities.. Eur J Med Chem.

[43] Rao MEB, Rajurkar VG (2011). Synthesis and biological studies of N-phenyl substituted 2-(5-(pyridin-4-yl)-1,3,4-oxadiazol-2-ylthio)acetamides.. Asian J Chem.

[44] Liu F, Luo XQ, Song BA, Bhadury PS, Yang S (2008). Synthesis and antifungal activity of novel sulfoxide derivatives containing trimethoxyphenyl substituted 1, 3, 4-thiadiazole and 1, 3, 4-oxadiazole moiety.. Bioorg Med Chem.

[45] Joshi S, Karnik AV (2002). Facile conversion of acyldiithiocarbazinate salts to 1,3,4-oxadiazole derivatives under microwave irradiation.. Synthetic Commun.

[46] Mission NAD, Doyle WC, Kans L (1969). Combating unwanted vegetation with 2-aryl-5- substituted 1,3,4-oxadiazoles.

[47] Oreste T, Mario G (1963). 2-Mercapto-5-heterocyclic-substituted-1,3,4-oxadiazoles.. Annali di Chimica.

[48] Pramanik SS, Mukherjee A (1998). Synthesis and in-vitro serotonin-3-antagonist activities of some new 1,3,4-oxadiazole-2-thiones.. J Indian Chem Soc.

[49] Frecentese F, Fiorino F, Perissutti E, Severino B, Magli E (2010). Efficient microwave combinatorial synthesis of novel indolic arylpiperazine derivatives as serotoninergic ligands.. Eur J Med Chem.

[50] Dini S, Caselli GF, Ferrari MP, Giani R, Clavenna G (1991). Heterogenity of [^3^H]-mepyramine binding sites in guinea pig cerebellum and lung.. Agents Actions.

[51] Khisti RT, Mandhane SN, Chopde CT (1998). The neurosteroid 3α-hydroxy-5α-pregan-20-one induces catalepsy in mice.. Neurosci Lett.

